# Maternal speech decreases pain scores and increases oxytocin levels in preterm infants during painful procedures

**DOI:** 10.1038/s41598-021-96840-4

**Published:** 2021-08-27

**Authors:** Manuela Filippa, Maria Grazia Monaci, Carmen Spagnuolo, Paolo Serravalle, Roberta Daniele, Didier Grandjean

**Affiliations:** 1 Swiss Center of Affective Sciences, Faculty of Psychology and Educational Sciences, University of Geneva, Geneva, Switzerland; 2 Department of Social Sciences, University of Valle D’Aosta, Aosta, Italy; 3 Maternal and Child Department, Parini Hospital, Aosta, Italy; 4 Department of Clinical Pathology, Parini Hospital, Aosta, Italy

**Keywords:** Health care, Paediatrics, Preterm birth, Psychology, Human behaviour

## Abstract

Preterm infants undergo early separation from parents and are exposed to frequent painful clinical procedures, with resultant short- and long-term effects on their neurodevelopment. We aimed to establish whether the mother’s voice could provide an effective and safe analgesia for preterm infants and whether endogenous oxytocin (OXT) could be linked to pain modulation. Twenty preterm infants were exposed to three conditions—mother’s live voice (speaking or singing) and standard care—in random order during a painful procedure. OXT levels (pg/mL) in saliva and plasma cortisol levels were quantified, and the Premature Infant Pain Profile (PIPP) was blindly coded by trained psychologists. During the mother’s live voice, PIPP scores significantly decreased, with a concomitant increase in OXT levels over baseline. The effect on pain perception was marginally significant for singing. No effects on cortisol levels were found. The mother’s live voice modulated preterm infants’ pain indicators. Endogenous OXT released during vocal contact is a promising protective mechanism during early painful interventions in at-risk populations.

## Introduction

Preterm birth rates are continuously increasing in almost all countries, with 15 million premature infants being born every year worldwide^1^. Despite rapid advances in technology, the number of preterm-born children who show short- and long-term sequelae of prematurity, even before reaching school age, remains high^2^. Around 40% of low birth weight preterm infants experience a complex spectrum of unfavourable neurodevelopmental outcomes^3^ when compared with their pairs at term. Thus, prematurity is of great concern for health policies in both low- and high-income countries^4,5^. The impaired development of preterm infants is not only associated with medical factors, but it is also at least partly a consequence of the atypical early-life environment of these infants^3^, including exposure to pain and separation from the primary caregivers^6–8^.

### Separation and pain in the Neonatal Intensive Care Unit (NICU)

The effects of separation and pain on premature infant’s development have been at the core of several research studies^9^. Very preterm infants experience early and prolonged separation from their parents during hospitalisation in the NICU, and this separation has profound impacts on their stress levels^8^, as well as on their autonomic, neuroendocrine and immune systems^10^.

This separation can also alter preterm infants’ long-term neurodevelopment, with important effects on emotional and attachment processes for both the infants and their parents^11–15^. Indeed, in the long term, preterm infants are an at-risk population with higher levels of stress, decreased resilience in coping with difficulties, and less secure attachment than their peers born at term^11^. A compromised attachment and bonding experience between mothers and their very preterm infants was associated with less intimacy with the infant and with difficulties in regulating socio-emotional stress at 3 months of age^16^.

Several painful clinical procedures occur for preterm infants in the first days after birth in NICUs in the context of partial or total separation from their parents. Since these procedures occur at a time of heightened sensitivity and rapid neurodevelopment, they might alter, in addition to later pain sensitivity, the brain structure and function of the infants^17^. Moreover, long-term dysfunctions in the neuroendocrine system and down-regulation of the hypothalamic–pituitary–adrenal axis, with lower cortisol levels, are associated with repeated painful procedures in preterm infants in the NICU^18^.

Although several epidemiological studies have reported advances in reducing procedural pain and improving the use of analgesic drugs in everyday clinical practice in NICUs^19–22^, there are consistent disparities in the administration of these analgesics to preterm infants among various countries and among various hospital units within the same country^22^. Moreover, recent advances in evidence-based studies demonstrate the ambivalence towards the use of drug analgesia techniques in the preterm population and the need to increase research on non-pharmacological forms of analgesia^23^. Although the role of OXT in the experience of pain has been documented^24^, its underlying mechanisms are still under debate and the protective impact that increased maternal care could have on OXT—and thus on pain perception in preterm infants—has never been investigated.

### Parental modulatory effect on infants’ pain

The positive effects of parental presence on pain modulation are still a matter of discussion, especially very early after birth. Although Piira and colleagues, in a systematic review^25^, affirmed that parental presence may not have a clear, direct influence on child distress, several research findings support the positive impact of parental contact on infants during noxious stimulation^26^. In particular, the efficacy of breastfeeding and skin-to-skin contact are accepted as alleviating pain responses during painful procedures^27–29^. The odour of the mother’s milk also appears to reduce pain from a heel stick in full-term neonates^30^ and preterm infants^31^, as well as pre-procedural pain^22^. Cortical pain responses after venepuncture in preterm infants has recently been investigated and the type of contact that the infant has with the mother has been shown to modulate neonatal brain processing of noxious stimuli^32^.

Understanding the mechanisms that modulate the effects of parental contact on pain perception in neonates could also provide insight into pain learning and protective actions against repeated pain exposure that can be enhanced in the neonatal period.

Parents in the NICU can modulate their preterm infant’s state and behaviour not only with skin-to-skin contact, but also with the medium of vocalisations. Early vocal contact is an early intervention that actively involves parents in emotional and meaningful vocal contact with their preterm infants during hospitalisation in the NICU^33^. It sustains the preterm infant’s physiological stability, with a significant decrease in critical events such as bradycardia, apnoea, and hypoxia, and it increases the occurrence of calm awake states^34^. When preterm infants in the NICU are exposed to more adult voices, they show significantly higher language and cognitive scores at 7 and 18 months^35^. In contrast, low parental presence and, consequently, decreased interaction and lower language exposure, contribute to the sensory deprivation experienced by infants in neonatal units, with impacts on their brain structure and neurodevelopmental outcomes^36^.

In two studies, using microanalytic methods, Filippa and colleagues illustrated the impact of live maternal vocalisations, both singing and speaking, on preterm infants’ behaviours^37^. Similarly, two specific infant pro-social behaviours, eye opening and smiles, are associated with an increase in the emotional content in the mother’s voice^38^ and with specific acoustical characteristics associated with infant-directed speech and singing^39^. Father’s vocal contact, like the mother’s voice, also has an impact on the preterm infant’s behavioural organisation and state, with calm awakening effects^40^.

### The key role of OXT

The role of OXT seems to be crucial, both in the paradigm of separation versus reunion of young infants with their mothers and in the inflammatory effects related to pain and stress^41,42^. OXT is released not only during affiliative interactions^43^, but also during social vocalisations^44^. The modulation of this oxytocinergic system is also correlated with positive effects on social behaviours^44,45^, pain^46^, stress due to separation^47^ and anxiety^48–50^.

In animal models, the periods of separation between mothers and offspring lead to reduced maternal care and a concomitant alteration in the regulation of OXT and vasopressin^51^. Early maternal separation interferes with the healthy development of OXT receptors in specific forebrain regions^52^. Offspring that received less maternal licking and grooming from low sensitive mothers exhibited associated changes in hypothalamic regions implicated in hormonal release and then indirectly related to maternal care^53^; they also showed reduced OXT receptor protein levels in the medial preoptic area, the lateral septum, the bed nucleus of the stria terminalis, the paraventricular nucleus of the hypothalamus, and the central nucleus of the amygdala^54^, providing a potential mechanism for the intergenerational transmission of individual differences in maternal behaviour.

From a protective perspective, in contrast, the provision of appropriate maternal care increases OXT levels in infants, and this affects brain organisation early in life in both animals and humans^55–58^. In particular, it has been shown that positive maternal care behaviours (1) increase OXT receptor binding in brain areas central to parenting and emotional behaviours, such the amygdala and the dorsolateral prefrontal cortex^59^, and (2) increase the reward that parents derive from their infants^60^.

Early tactile care behaviours, such as skin-to-skin contact in the newborn period, increase OXT levels in both mothers and infants^61,62^, decrease salivary cortisol reactivity and improve salivary cortisol concordance between mother and infant^12^. Interestingly, OXT receptors are present in the peripheral terminal axons of the skin^63^, and touch-evoked OXT release could also explain the analgesia induced by tactile stimulation^64^.

As regards the role of OXT in pain experience it has been suggested that in the animal model OXT has precise functions in the physiological responses to pain and stress^65^. In rodents, OXT has analgesic effects acting on pain matrix structures^66^. In particular, endogenous oxytocin exerts an analgesic action, for example in newborn pups with a reduction of the depolarizing action of GABA on nociceptive neurons^67^.

In humans, OXT acts on brain regions involved in pain processing^68^, but the mechanisms linking OXT and pain perception in humans are not yet fully understood^66^. Results seem sometimes controversial, but generally promising, suggesting that OXT may decrease pain sensitivity^69^. OXT reduces visceral pain symptoms in patients with irritable bowel syndrome^70^, low back pain after intrathecal infusion^71^, and its intranasal administration diminishes headache^72^. Additionally, OXT modulates the emotional dimension of pain expression^73,74^. These observations and others led to the proposal that OXT modulates several dimensions of pain expression, and has strong effects on emotional output, attentional processes, and social interactions^75^.

Finally, it is known that OXT and cortisol are both implicated in the effects of pain and that OXT can inhibit the function of the hypothalamic–pituitary–adrenal axis at several levels during the production of cortisol^76,77^. It is also known that basal cortisol secretion is altered in preterm infants^78^, especially in infants born at low gestational age^79^, who were exposed to the most invasive procedures during neonatal care in addition to being developmentally the most immature.

In the present study, we aimed to evaluate the effects of reducing separation between the mother and her prematurely-born offspring on pain perception and on OXT release during a routine painful clinical procedure in the NICU. We predicted that early vocal contact would reduce the signs related to preterm infant pain by affecting the oxytocinergic system, generating an increase in endogenous OXT levels correlated with a decrease in infant pain signs.

## Methods

### Study design and participants

The single­centre study was conducted in a level II NICU at the Parini Hospital (Aosta, Italy). A total of 68 preterm infants were born in the NICU hospital between January 2018 and April 2019; of these, 47 did not meet the inclusion criteria, their length of stay in the NICU was expected to be less than 48 h, or they were not included because of technical or logistic problems. Infants were assessed for eligibility by a senior clinician; 21 of them met the inclusion criteria, were medically stable and were approached for enrolment and were enrolled. However, one was excluded because of incomplete data. Twenty preterm infants were thus recruited.

The final sample met the following inclusion criteria: (1) age > 29 weeks gestational age, (2) weight > 1000 g and (3) stable medical condition. Preterm infants who needed mechanical ventilation and additional oxygen and who had specific pathological conditions (i.e., genetic malformation, presence of periventricular or intraventricular haemorrhage, periventricular leukomalacia) were excluded from the study, as were mothers with a history of substance abuse or with mental health problems. The data for the mothers and newborns are summarised in Table 1.

**Table 1:** Infant demographics.

	Mean *(SD, range)*
**Characteristics at birth**	
Gestational age at birth (weeks)*	32.7 *(9, 6 days)*
Birthweight (g)	2042 *(392, 5)*
Apgar score at 1 min	7.05 *(1, 5)*
Apgar score at 5 min	8.1 *(1)*
Mode of delivery	Spontaneous vaginal delivery (40%)
	Cesarean Sect. (60%)
Sex	Female (45%)
Citizenship	Italian (100%)
Primiparous	Yes (90%)
Mother’s age	29.2 *(4, 62)*
*Characteristics *at time of clinical procedure	
Gestational age at test (weeks)	34.8 *(10 days)*
Postnatal age at test (days)	3 *(range 1–8)*
Weight (g)	2264 *(325)*
Number of painful procedures before testing	7 *(range 3–15)*
Phototherapy before testing	Yes (70%)

*Postmenstrual age should be used for clarity in neonatal practice. In our unit, the infants' age is recorded in the medical and nursing notes as gestational age, and we have used this terminology.

At discharge, all participants had passed the AABR bilateral hearing screening test. The official Hospital Ethical Committee of Aosta reviewed and approved the study (I.C. n. 90.513; date of approval: 10/20/2017), written informed parental consent was obtained and all experiments were performed in accordance with relevant guidelines and regulations.

### Procedure

Electronic data capture of heart rate, respiratory rate and oxygen saturation, as well as video recordings, began at least 5 min before the clinical procedure in order to establish a baseline of clinical stability for every infant (baseline duration: 5 min). Each infant was recorded on three test occasions. During the intervention, mothers were asked to speak or to sing to their preterm infants in the incubators for 5 min preceding the heel prick procedure and for the subsequent 5 min.

**Figure 1:**
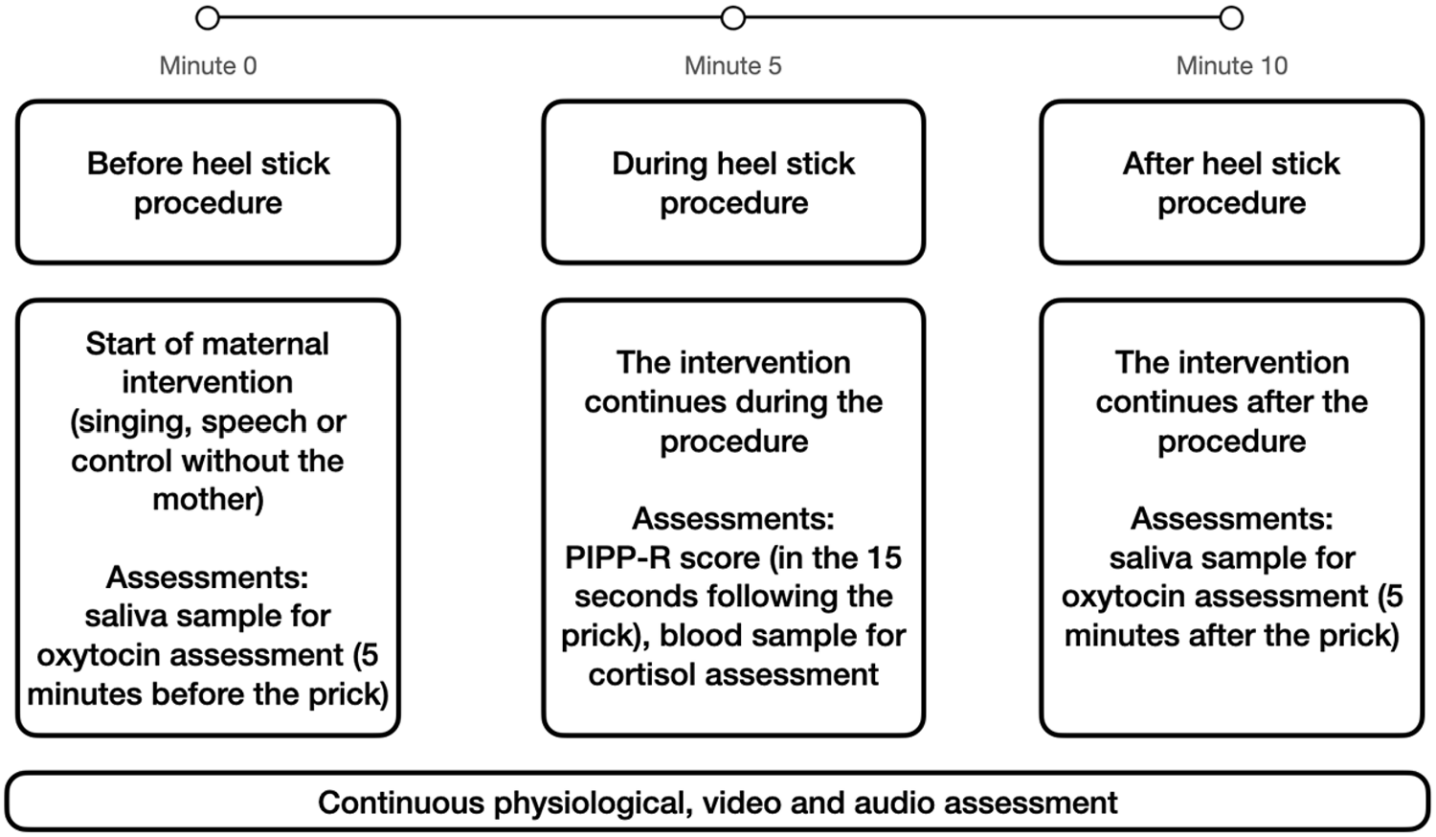
Procedure diagram of the sample collections, assessments and intervention.

During the control condition (without the mother), the newborn was placed by the nurse in the incubator in the standard care conditions recommended for painful procedures (supine position, wrapped and contained by the nest). During the intervention and control conditions, physiological and video data were continuously captured. See Fig. 1 for procedure diagram of sample collection, assessments and intervention.

In both intervention conditions, speaking and singing, the mothers were asked not to touch the baby but to pay close attention to his/her reactions and to modulate the voice accordingly. A nurse was present during all procedures.

Before each intervention, the background noise levels were acquired via a calibrated sound level meter (Voltcraft Phonometer SL-10; Conrad Electronic, Hirschau, Germany) in the room where the intervention took place, inside the incubator, and 20 cm from the newborn’s head. This measure was assessed at every intervention session to ensure that the mother’s voice was audible to the newborn (i.e., that it exceeded the background noise of 10 dBA)^80^ in order to support 100% speech and song intelligibility among mothers and preterm newborns. Moreover, mothers were instructed not to exceed the recommended levels for sound pressure^81^ in order to prevent overstimulation.

Finally, they were instructed to open the incubator window, to speak and to sing through the window, and to keep a distance approximately of 20 cm from the newborn’s head. The correct position was constantly verified by the researcher.

No other specific instructions were given to the mothers, who could use their intuitive parenting behaviours in proximity to the babies. No further analyses were performed on these values.

Testing was performed over three consecutive days in the first days of extra-uterine life (for details on postnatal days, see Table 1). The order of the stimuli (speaking or singing) and the control condition was randomly selected over the three testing days across the infants. The randomisation was performed by using a secure web-based randomisation system, through which the study investigator registered new patients and obtained the treatment arm assignment.

### Outcome measures

The co-primary outcome measures were a behavioural pain score calculated after the heel prick with the Premature Infant Pain Profile–Revised (PIPP)^82^ and salivary OXT levels.

#### PIPP score

The PIPP score was calculated in the 15-s period after the heel prick procedure by both a trained nurse who was observing the procedure and offline by two blinded independent and trained coders on muted video tracks. The PIPP score is a cluster of physiological and behavioural measures. Physiological assessment was calculated on the heart rate and oxygen saturation levels as collected from the patient monitor by the researcher. Inter-rater reliability was assessed by three independent coders: expert coders 1 and 2 performed blinded ratings from offline muted videos and digitally recorded physiological parameters, whereas coder 3 was a trained nurse and performed a direct online rating of the PIPP scores. The three independent raters achieved a high degree of reliability, as confirmed by Spearman’s correlation analysis, with values of 0.73 for coder 1, 0.78 for coder 2, and 0.70 for coder 3 when compared with the mean of the PIPP scores obtained on all raters.

#### OXT measure

Saliva samples were collected without stimulants by using an absorbent device^83^ that was placed in the newborn’s mouth and then centrifuged and stored at − 20 °C until analysis. Saliva samples were collected twice, 5 min before and after the heel prick procedure, in the three conditions. Salivary OXT concentrations (pg/mL) were quantified by radioimmunoassay (RIAgnosis, Munich, Germany). A quantity of 300 μL of saliva was evaporated for each sample (SpeedVac, Thermoscientific Inc, Waltham, MA, USA); 50 μL of assay buffer and 50 μL antibody were then added. After a 60-min preincubation interval, an additional 10 μL of 125 I-labelled tracer (PerkinElmer, Waltham, MA, USA) was used and samples incubated for 3 days at 4 °C. The detection limit was fixed at 0.5 pg/sample range, depending on the age of the tracer, with typical displacements of 20–25% at 2 pg, 60–70% at 8 pg, and 90% at 32 pg of standard neuropeptide. The intra- and inter-assay variabilities were < 10%. When the amount of saliva was insufficient, saliva samples from several infants during the same condition were pooled, and serial dilutions of saliva samples containing high levels of endogenous OXT were run strictly parallel to the standard curve indicating immuno-identity. Single, not pooled, saliva analysis was performed for 73% of the collected samples.

#### Cortisol measure

Blood samples were collected during the heel prick procedure, centrifuged 10 min at 2000 rpm, and each serum separated from the clot and frozen at − 20 °C until analysis. Cortisol concentrations were measured by using the chemiluminescent immunoassay method on a DiaSorin Liaison XL analyser (Saluggia, Italy) according to the manufacturer’s instructions. Serum samples were analysed in different batches; however, all samples from each newborn were always assayed in the same batch.

### Statistical analysis

General linear mixed models in R software (version 2.15.0) were used. R Studio (version 0.97.551) was applied instead of classic analysis of variance in order to include random factors (e.g., pooled samples, identity; Team, 2013). To test the significance of the different experimental conditions, we systematically used chi square tests for the comparison of alternative models (e.g., a model with main effects compared with a model that also included the interaction). The following fixed effects factors were specified: the three Conditions (during mother’s singing [Singing], during mother’s speech [Speaking], and without mothers [Control]) and the Time factor (Pre and Post conditions for OXT levels). The random factors included in our models were dyad ID, and pooled or not pooled samples. Our dependent variables were cortisol levels, OXT levels and PIPP scores.

For cortisol analysis, we identified the outliers for values > 22.15, i.e., higher than double the standard deviation of the mean of the total conditions. In the analysis, we substituted the missing values (5%) with the mean of the same conditions (6.4339 for control; 6.6927 for singing; 7.6883 for speaking).

## Results

**Figure 2:**
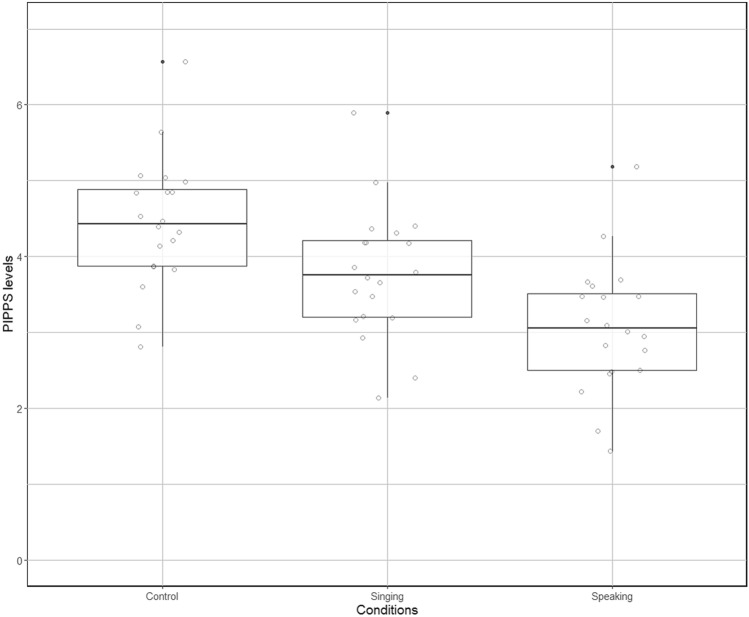
Preterm infants Pain Profile scores presentation in the Control, Singing and Speaking conditions during painful procedures.

The statistical model that included Condition (Singing, Speaking, and Control modalities) explained significantly more variance of the PIPP score than did a simpler model with only random effects (IDs and Pooled/NoPooled; χ^2^ (2) = 7.16, *p* = 0.028). Planned contrasts revealed that preterm infants’ PIPP scores were significantly lower in the Speaking condition than in the Control condition in the absence of the mother’s voice (χ^2^ (1) = 7.45, *p* = 0.006). No significant differences were found for the Singing condition for the same comparison (χ^2^ (1) = 1.76, *p* = 0.18; see Fig. 2).

**Figure 3:**
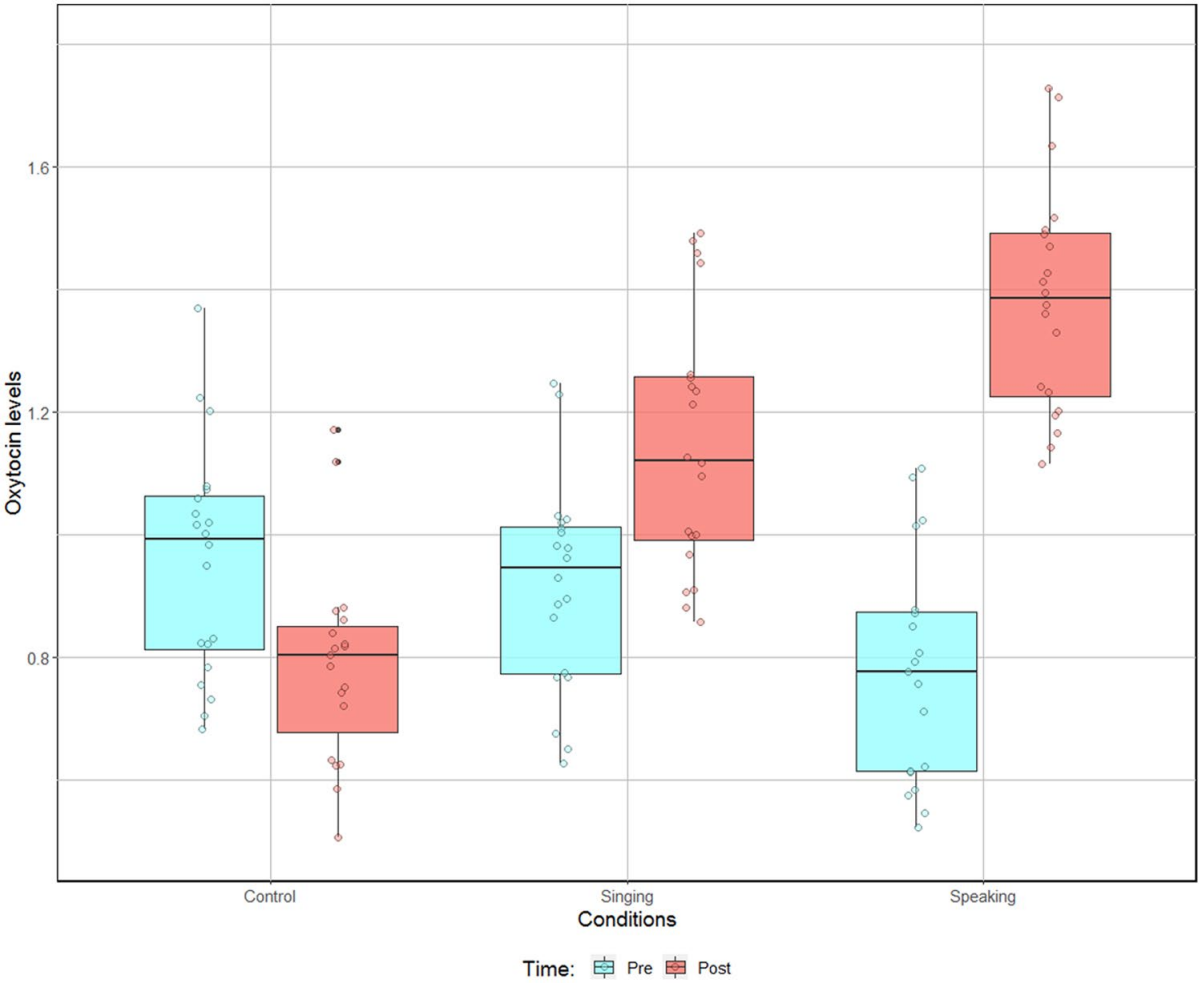
Preterm infants’ oxytocin levels (pg/mL) before and after painful procedures in the Control, Singing and Speaking conditions.

OXT analysis revealed that the statistical model that included the planned interaction of Condition (Singing, Speaking, and Control modalities) with Time (Pre and Post modalities) explained significantly more variance of OXT levels than did the model with main effects of both Condition and Time (χ^2^ (2) = 6.99, *p* = 0.03). Note that the comparisons of models for each main effect of Condition and Time were not significant (χ^2^ (2) = 1.79, *p* = 0.41, and χ^2^ (1) = 2.87, *p* = 0.09, respectively). Planned contrasts revealed (1) no significant differences for the Pre modality of the Time factor between the three Conditions (Control vs. Singing: χ^2^ (1) = 0.06, *p* = 0.80; Control vs. Speaking: χ^2^ (1) = 0.91, *p* = 0.34; Singing vs. Speaking: χ^2^ (1) = 0.49, *p* = 0.48); (2) significant differences for the Post modality of the Time factor between Speaking and Control modalities of the Condition factor (χ^2^ (1) = 7.72, *p* = 0.005); (3) marginally significant differences between Control and Singing (χ^2^ (1) = 2.91, *p* = 0.088); and (4) no significant differences between Singing and Speaking (χ^2^ (1) = 1.15, *p* = 0.28; Fig. 3). Note that the Pre vs. Post comparisons confirmed the significant increase of OXT levels for the Speaking modality (χ^2^ (1) = 7.99, *p* = 0.0047), whereas the same contrast did not reach significance for both the Control and the Singing modalities (χ^2^ (1) = 0.82, *p* = 0.37, and χ^2^ (1) = 1.11, *p* = 0.29, respectively). This analysis was not significantly affected by the infant’s sex (χ^2^ (1) = 0.13, *p* = 0.71).

The cortisol levels showed no significant differences when we included the Condition factor compared with a model with only the random factors (χ^2^ (2) = 2.17, *p* = 0.34). Spearman correlation analysis was performed and no significant correlation between cortisol levels and OXT levels in preterm infants was found for the Pre condition (r_s_ =  − 0.06, *p* = 0.65, *N* = 60) and the Post condition (r_s_ = 0.07, *p* = 0.59, *N* = 60). Similarly, no significant correlation between cortisol levels and PIPP scores was found (r_s_ =  − 0.01, *p* = 0.93, *N* = 60).

## Discussion

The main aims of this study were to investigate how mothers’ interventions through vocalisations (speaking and singing), compared with a control condition without the mother, could modulate (1) the signs of pain in the preterm infants, (2) the endogenous amount of OXT in the preterm infants’ saliva samples, and (3) the level of cortisol in their plasma samples after acutely painful clinical procedures. The results showed that maternal infant-directed speech has a beneficial effect on preterm infants’ pain, as evidenced by a significant decrease in PIPP scores. Furthermore, OXT levels significantly increased during the mother’s speech, but only marginally for singing, when compared with the absence of the mother. No effects of the three conditions—speaking, singing or standard care—on infants’ plasma cortisol levels were found.

To our knowledge, this is the first study to not only provide the preliminary data on the potential analgesic properties of the live maternal voice during painful procedures in preterm infants, but also to show that endogenous OXT regulation could act as a potential protective mechanism for early pain perception. Further data based on preclinical animal models are needed to demonstrate the causality link between maternal vocalizations, OXT levels and pain perception. The administration of an OXT receptor antagonist to offspring during a painful procedure, in the presence of maternal vocalizations, could show the existence of a causal link. These preliminary results extend previous studies of infants conducted in an animal model, which showed that early relational experiences can persistently affect social behaviours by modifying the OXT system^55^. In particular, it is known that experiences of early contact have long-term effects—even intergenerational effects^84^—through the modulation of the OXT system. Although singing was associated with an increase in OXT, only in the speaking condition were significant correlations demonstrated. The OXT distributions are often heterogeneous and this may explain the marginal effects for the singing condition in a small population.

Early and repeated painful experiences (here especially in preterm infants) induce long-term over-sensitisation to pain and stress, and have significant consequences for infant social and emotional competencies^7^. Concerning maternal separation, the OXT system might also play a crucial role in repairing and reconstructing the infant’s resilience in response to painful stimuli.

One of the main aims of pain management in neonates is to maximise the their capacity to cope with and recover from painful experiences^85^. If the present results are confirmed through studies that investigate pain responses in preterm infants at the brain level and through robust animal models in which OXT antagonists are manipulated during painful procedures, new early protective interventions can be designed for preemies in the NICU. Parental vocalizations should thus be encouraged during all phases of painful procedures in the NICU, including the preparation phase, the acute pain phase and the consolatory phase following the procedure.

The active involvement of parents in the early care of preterm infants is one of the primary goals of infant- and family-centred developmental care^86^. Positive social and emotional interactions between parents and infants in the NICU have long-term effects not only on infants, but also on parent mental health outcomes^87^, reducing parental depression and anxiety, and reinforcing the parental role.

The present preliminary results, if supported by further studies and in line with early family-centred interventions, could suggest that maternal direct vocalizations should be integrated in standard care in the NICU during infants’ painful and stressful experiences.

## Study limitations

One limitation of the present study is the small sample size, although this vulnerable population needs individualized studies in order to design future preventive health interventions. Several difficulties led to our study having a small sample size, including the vulnerability of the preterm population and the active involvement of parents during painful procedures, which is not part of standard care in an NICU. Future research is needed to enlarge the sample size, both in number and types of involved patients. Fragile very preterm infants, born at less than 32 weeks of gestational age, and newborn infants requiring painful procedures during hospitalization should be included in future studies. In the present study, only maternal vocalizations were manipulated, but the introduction of a control condition with a silent maternal presence is recommended for future studies, in the absence of painful procedures^88^. Another limitation of the study is the blood sample for cortisol analysis. In order to avoid an additional painful procedure for research purposes, we took additional blood during the same procedure. Thus, we had no possibility of analysing the cortisol changes in blood samples at different time points, i.e., after 5 and 10 min following the procedure. Finally, it is known that OXT distribution is heterogeneous, especially in very small saliva samples such as those for preterm infants. However, the existing correlations between OXT levels and PIPP scores are preliminary promising results. Future studies, robust preclinical research and investigations into preterm infant pain through neuroimaging techniques are needed to replicate the present results.

## Conclusion

The universal right to pain relief, especially in vulnerable patient populations, is undeniable^89^. The non-noxious perspective in protective medicine should be at the core of future research, and the search for alternative, safe and effective pain management must be a primary concern of researchers and of science.

We believe that our study is a starting point for further investigations into the role of maternal vocalizations as a protective factor for preterm infants against the effects of pain and separation during hospitalisation in the NICU. The specific role of endogenous OXT is a promising mechanism of action for early protective intervention in at-risk populations, who are too often exposed to pain, stress and separation from their caregivers during hospitalisation.
